# Assessing Predicted HIV-1 Replicative Capacity in a Clinical Setting

**DOI:** 10.1371/journal.ppat.1002321

**Published:** 2011-11-03

**Authors:** Roger D. Kouyos, Viktor von Wyl, Trevor Hinkley, Christos J. Petropoulos, Mojgan Haddad, Jeannette M. Whitcomb, Jürg Böni, Sabine Yerly, Cristina Cellerai, Thomas Klimkait, Huldrych F. Günthard, Sebastian Bonhoeffer, the Swiss HIV Cohort Study

**Affiliations:** 1 ETH Zürich, Institute of Integrative Biology, Zürich, Switzerland; 2 Princeton University, Department of Ecology and Evolutionary Biology, Princeton, New Jersey, United States of America; 3 Division of Infectious Diseases and Hospital Epidemiology, University Hospital Zurich, University of Zurich, Zurich, Switzerland; 4 Monogram Biosciences, South San Francisco, California, United States of America; 5 Swiss National Center for Retroviruses, Institute of Medical Virology, University of Zurich; Zurich, Switzerland; 6 Laboratory of Virology and AIDS Center, Geneva University Hospital, Geneva, Switzerland; 7 Division of Immunology and Allergy, Centre Hospitalier Universitaire Vaudois and University of Lausanne, Lausanne, Switzerland; 8 Institute of Medical Microbiology, Department Biomedicine, University of Basel, Basel, Switzerland; NIH/NIAID, United States of America

## Abstract

HIV-1 replicative capacity (RC) provides a measure of within-host fitness and is determined in the context of phenotypic drug resistance testing. However it is unclear how these in-vitro measurements relate to in-vivo processes. Here we assess RCs in a clinical setting by combining a previously published machine-learning tool, which predicts RC values from partial pol sequences with genotypic and clinical data from the Swiss HIV Cohort Study. The machine-learning tool is based on a training set consisting of 65000 RC measurements paired with their corresponding partial pol sequences. We find that predicted RC values (pRCs) correlate significantly with the virus load measured in 2073 infected but drug naïve individuals. Furthermore, we find that, for 53 pairs of sequences, each pair sampled in the same infected individual, the pRC was significantly higher for the sequence sampled later in the infection and that the increase in pRC was also significantly correlated with the increase in plasma viral load and with the length of the time-interval between the sampling points. These findings indicate that selection within a patient favors the evolution of higher replicative capacities and that these in-vitro fitness measures are indicative of in-vivo HIV virus load.

## Author Summary

Determining how well different genotypes of HIV can replicate within a patient is central for our understanding of the evolution of HIV. Such in vivo fitness is often approximated by in vitro measurements of viral replicative capacities. Here we use a machine-learning algorithm to predict in vitro replicative capacities from HIV nucleotide sequences and compare these predicted replicative capacities with clinical data from HIV-infected individuals. We find that predicted replicative capacity correlates significantly with the concentration of HIV RNA in the plasma of infected individuals (virus load). Furthermore, we show that the predicted replicative capacity increases in the course of an infection. Finally, we found that the temporal increase of replicative capacity correlates significantly with the temporal increase of virus load within a patient. These results indicate that (predicted) replicative capacity is a useful measure for viral fitness and suggest that virus genetics determines virus load at least to some extent via replicative capacity.

## Introduction

Measuring the fitness of HIV-1 is notoriously difficult. At the between-host level, fitness can be interpreted as the transmission potential which is defined as the expected number of transmissions in the course of an infection [1]. This quantity can however only be measured in cohorts of untreated patients with known infection status that are followed over long time periods [1]. At the within-host level, fitness is determined by the average number of secondary infected cells resulting from a single infected cell in vivo. This hypothetical quantity is difficult to determine [2] but can be approximated by in-vitro measurements of the replicative capacity (RC) (see [3]). However, the in-vivo relevance of such in vitro fitness values is largely unclear.

In a recent publication, some of the authors of this article described a computational method to predict RC values on the basis of viral amino-acid sequences [3]. To this end, a machine-learning algorithm based on a quadratic fitness model was applied to a training data set of 65,000 amino-acid sequences of the pol gene and the associated RC values. The resulting RC-predictor could explain roughly 40% of the deviance of RC values in a test-data set consisting of 5,000 sequences, which had not been used for the inference of this predictor. In the present study, we apply this computational predictor to clinical data from the Swiss HIV Cohort Study (SHCS) (www.shcs.ch) in order to obtain an assessment of the RC-predictor in an independent dataset and to study its correlation with plasma HIV RNA viral load, a known surrogate marker associated with disease progression [3].

## Methods

### Ethics statement

The Swiss HIV cohort study was approved by individual local institutional review boards of all participating centers (www.shcs.ch). Written informed consent was obtained for each SHCS study participant.

### RC-prediction

Fitness is measured as the log replicative capacity of HIV-derived amplicons [representing all of Protease(PR) and most of Reverse Transcriptase (RT)] inserted into a constant backbone of a resistance test vector. The models are then trained to predict this fitness from the amino-acid sequence of the amplicons. Details on the experimental measurement of the RC values and on inferring the predictor have been published in [3]. Here, we briefly reiterate the principles of the models fitted.

In essence, the predictor is based on fitting the data consisting of amino acid sequences s and the corresponding log-RC values (*w*) with the following model 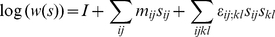
(M1)
*s_ij_* denotes the presence (*s_ij_* = 1) or absence (*s_ij_* = 0) of allele j at position *i.* (or more generally, if an ambiguity in the population sequencing is consistent with several amino acids at a given position, *s_ij_* denotes the probability of allele j at position i). The model parameters *I*, *m*_ij_ and *ε*_ij;kl_ can be interpreted as intercept, main effects, and epistatic effects. As the number of parameters exceeds the number of data-points, the model *M1* has been fitted to the data on the basis of a machine learning approach (generalized kernel ridge regression). With this approach over-fitting is no concern because the sub-dataset on which the predictor is evaluated is independent from the sub-dataset from which the predictor is inferred (see supplementary material of Hinkley et al. [3] for a detailed description of the fitting procedure).

### Clinical and sequence data

We assessed the RC-predictor by using two datasets collected from untreated, chronically infected patients. The latter criterion was introduced because HIV RNA levels are usually very high during acute HIV infection, and it was ensured by discarding data points measured within the first 180 days after the first positive HIV test. The patients were enrolled in the Swiss HIV Cohort Study, a longitudinal multicenter observational cohort study (SHCS) (www.shcs.ch) [4]. These datasets consist of clinical data (Table 1) and the corresponding viral amino acid sequences from the SHCS drug resistance database [5]. We focus on patients, for whom amino-acid sequences of the entire protease and the first 303 amino acids of the reverse transcriptase were available. We only consider sequences, which have been obtained from therapy-naïve patients infected with HIV-1 subtype B because the training set originated solely from subtype B strains. The first set consists of nucleotide sequences with the corresponding HIV RNA virus load measurements (plasma viral load set; n = 2073 patients). Selection of viral load measurements is restricted to values obtained within 30 days before or after the genotypic tests, but before initiation of antiretroviral therapy. The second set contains 53 patients for whom genetic sequences are available at two time points, which are at least 6 months apart (median [interquartile] distance between the two measurements: 3.9 [1.9; 7.4] years; longitudinal set) (see [6] for more details on this dataset).

**Table 1:** Multivariable regression model to assess the association of log10 HIV RNA load with the predicted replicative capacity.

	N (%)^a^	Regression Coefficient [95% Confidence Interval] b	P-value
Median [IQR] c estimated replicative capacity	0.62 [0.40 to 0.81]	**0.29 [0.18 to 0.40]**	<0.001
Sex			0.02
Male	1685 (82.3%)	Reference	
Female	388 (18.7%)	**−0.12 [−0.22 to −0.02]**	
Median [IQR] age	37 [31 to 43]	0.02 [−0.02 to 0.06] d	0.242
Mode of HIV acquisition			<0.001
Heterosexual contacts	483 (22.3%)	**−0.20 [−0.29 to −0.11]**	
Homosexual contacts	1144 (55.2%)	Reference	
Intravenous drug use	446 (21.5%)	**−0.19 [−0.28 to −0.10]**	
Ethnicity			0.015
White	1925 (92.9%)	Reference	
Black	24 (1.2%)	**−0.41 [−0.66 to −0.16]**	
Hispanic	68 (3.3%)	−0.10 [−0.27 to 0.07]	
Asian	35 (1.7%)	−0.05 [−0.22 to 0.12]	
Other	21 (1.0%)	0.13 [−0.18 to 0.43]	
Sequence generating laboratory			0.087
A	215 (10.4%)	Reference	
B	420 (20.3%)	−0.05 [−0.19 to 0.09]	
C	1438 (69.4%)	**−0.12 [−0.24 to 0.01]**	
Median [IQR] year of sequence generation	2008 [2006 to 2008]	**−0.03 [−0.05 to −0.01] e**	
Median [IQR] CD4 counts/microliter at time of sampling for genotyping	298 [162 to 464]	not done^f^	
CD4 count groups (by 25th percentiles) f			<0.001
0 to 162	542 (25%)	Reference	
163 to 298	542 (25%)	**−0.41 [−0.50 to −0.32]**	
299 to 464	543 (25%)	**−0.63 [−0.72 to −0.53]**	
465 to 1522	541 (25%)	**−0.90 [−1.00 to −0.79]**	
Ever had CDC stage C event prior to genotyping	206 (9.9%)	0.10 [−0.01 to 0.21]	0.085

a unless stated otherwise.

b Regression coefficients printed in bold face are statistically significant at the 5% level.

c Abbreviations: IQR interquartile range.

d Regression coefficient per 10 years increase.

e Regression coefficient per year increase.

f because of better regression fit the final model included CD4 cell count as 4 categories.

### Statistical analyses

Relationships between HIV RNA and pRC were modelled by the use of univariable and multivariable linear regression. Model assumptions were verified by inspecting residual versus fitted plots and by checking for unequal variance across fitted values (heteroskedasticity) and outliers. Because these diagnostics suggested the presence of heteroskedasticity we performed “robust” versions of linear regressions, which estimate a weighted variance based on the Huber−White method.

Statistical calculations were carried out with Stata 11.2 (Stata Corp., College Station, TX, USA). The level of significance was set at 0.05, and all p-values are two sided.

## Results

Demographic and clinical characteristics of our study population are displayed in table 1. We assessed the predicted RC (pRC) with respect to two clinically relevant quantities or processes: Firstly, the relation between pRC and virus-load measurements measured around the same time and, secondly, the temporal change of pRC within ART-naive individuals.

**Figure 1:**
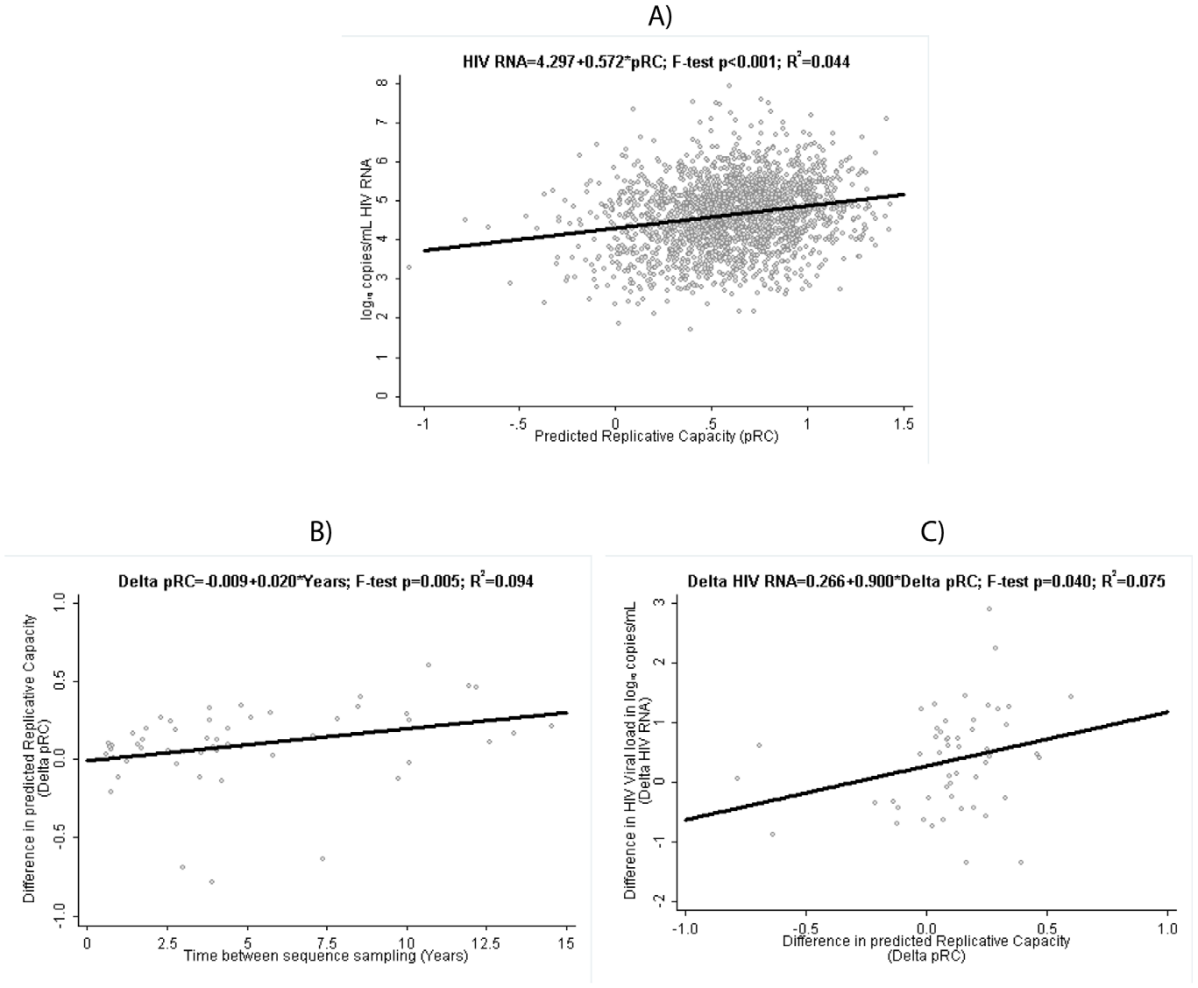
Clinical Relevance of predicted Replicative Capacity (pRC). (**A**) Relation between pRC and virus load (measured as log10(copies of RNA/ml)) in the RNA-load dataset. (**B**) Temporal increase of pRC in the Longitudinal Dataset: relation between time difference between sequence samples and the change in pRC. (**C**) Relation between change in pRC and change in RNA-load in the Longitudinal Dataset.

In the plasma viral load dataset (2073 patients), values for RC predictions (pRC) were ranging from −1.07 to 1.43 units (median [interquartile range] 0.62 [0.40; 0.81]), and corresponding median [interquartile] HIV RNA levels were 4.7 log10 copies/mL [4.1; 5.2]. Using univariable linear regression analysis, we find a highly significant effect of the pRC value on virus load (F−Test *p<*0.001; see Figure 1A): a 1 unit increase in pRC is associated with an 0.57 increase [95% confidence interval 0.45; 0.69] in log10 HIV RNA. The fraction of variance in virus load explained through the pRC (R^2^) is 4.4%. Although somewhat attenuated, this effect of pRC on virus load remains highly significant (*p<*0.001; 0.29 [0.18; 0.40] log10 copies/mL HIV RNA per 1 unit increase in pRC ;table 1) if we control in a multivariable regression model for age, ethnicity, risk group, sex, CDC C stage and CD4 count at time of viral sequencing, and the laboratory that generated the sequence data. The association between HIV RNA and pRC changes only minimally when the fully adjusted regression model is re-estimated on individuals without any evidence for transmitted drug resistance mutations as defined by the most recent WHO surveillance list [7] (n = 1909; regression coefficient [95% confidence interval] 0.30 [0.18; 0.42] log10 copies HIV RNA per unit change pRC).

For the longitudinal dataset, we find that the pRC value increases in the course of an infection. Among the 53 patients with two viral sequences available taken at least 6 months apart, the median [interquartile] difference in pRC is 0.10 units [0.04; 0.25] and is statistically significantly different from 0 (p sign rank<0.001). Unadjusted linear regression estimates this increase in pRC at 0.020 units per year [95% confidence interval 0.006; 0.035] (figure 1B). At the same time, HIV RNA also tended to be higher at the second, later time point, with a median of 0.42 log10 copies/mL [−0.28; 0.88] (sign rank p = 0.005). Consequently, we find a statistically significant association between the change in pRC correlates and the change in HIV RNA over time in these 53 patients when applying a linear regression model to the data, which predicts a rise of 0.90 [0.01; 1.79] log10 copies/mL in HIV RNA per 1 unit increase in pRC over time (figure 1C). This finding suggests that within-host evolution seems to be characterized by a trend towards higher replication rates, and consequently higher plasma HIV RNA viral loads.

The above analyses were based on untreated patients sampled after the acute phase of the infection. We find similar results if we exclude patients, which have been sampled in the AIDS phase (defined as patients with at least one CDC stage C event, n = 206). In particular, we still find a highly significant (p<0.001) correlation between pRC and RNA load (slope: 1 unit increase in pRC is associated with an 0.54 increase [95% confidence interval 0.41; 0.66] in log10 HIV RNA) and a significant (p = 0.0058) increase of RC over time (increase in pRC at 0.020 units per year [95% confidence interval 0.006; 0.035]). Only the significance-level of the correlation between the temporal change of pRC and the temporal change of RNA load changes from ‘significant’ (p = 0.04) to ‘trend’ (p = 0.058); however even in this case the point estimates for the regression coefficient are very similar in both cases (0.9[0.01; 1.79] vs. 0.84[−0.03; 1.70]).

## Discussion

How do the pRCs analyzed here relate to previous findings? For example, the 6 sequences (in our data-set) carrying the lamivudine mutation M184V, which has a large negative fitness effect on the virus [8] and has been associated with an 0.3 log10 copies lower HIV RNA relative to wild type [9], had a median [interquartile range] pRC of 0.1 [−1.3; 0.6], compared to 0.6 [0.4; 0.8] in the 1909 sequences without any transmitted resistance mutations (Wilcoxon rank sum p<0.001). Overall, the pRC varied over a range of 2.5 units from minimum to maximum. Our unadjusted and adjusted regression models would therefore predict a difference in HIV RNA of approximately 1.4 and 0.73 log10 copies/mL between the lowest and the highest pRC value. Yet HIV RNA viral loads varied over 6 logs from 1.9 to 7.9 log10 copies/mL in our dataset. This discrepancy is not very surprising given that our predictor for RC only takes the variation of 400 amino acid positions (roughly 10% of the genome of HIV) into account. However, the finding of a correlation of pRC and HIV RNA is robust, as confirmed by several sensitivity analyses, and it is consistent with a number of previous studies, which have also shown a correlation between in vitro measurements of RC and virus load [10], [11], [12], [13], [14].

Our findings thus support the notion that virus load is to a large extent controlled by virus genetics [15], [16], [17]. The fraction of variance explained by pRC (4.4%) is much lower than the fraction of variance in virus load explained by virus genetics in previous studies [15], [16], [17], but it should be borne in mind that the estimates of studies [15], [16], [17] are based on the variation in the entire genome (Note that this is the case even for Alizon et al.[15], because, even though the phylogenies used in that study were inferred from the pol-gene, they reflect the relatedness of the entire genome provided that recombination is not too common on an epidemiological level). It should also be noted that our results argue that at least a part of the virus' genetic control of the virus load established in patients appears to be mediated by the replicative capacity of the virus. This finding that virus load is controlled by RC contrasts the interpretation that virus load is mainly determined by the activation-rate of CD4 cells[18]. However, the relative importance of these different factors remains an open question. The increase of pRCs over time is also consistent with previous observations [19], and supports the view that, within a single host, HIV is selected for higher replicative capacities over time.

Overall our results show on the basis of a computational predictor, firstly that in vitro replicative capacity increases in the course of infection, which is consistent with the interpretation that RC is a determinant of fitness at the within-host level, and secondly that RC is linked to virus load, which has been shown to be a in vivo determinant of viral fitness at an epidemiological level [1]. In our view, it is remarkable that predicted RC based on partial pol sequences representing only 10% of HIVs genome correlates with virus load. Accordingly, taking into account the variation in the entire HIV genome (as will become possible in the future) may help to develop much more accurate predictors of virus fitness and virus load.
